# Cholera

**Published:** 1886-01

**Authors:** 


CHOLERA.

In Spain, up to the close of the week ending October 21st, 273,549 cases had been reported with a total mortality of 101.346. In Italy, there have been 1,375 cases with 689 deaths.

---

A Chicago doctor was about to anæsthetize a patient, when, in answer to a question, he informed the victim that he would be entirely unconscious, and know nothing until the offending growth had been removed. The patient accordingly commenced to fish his loose change out of his pocket. "Oh, you need not mind the fee until I am through," remarked the considerate doctor. "I don't intend to pay you yet," returned the patient; "I wish merely to count my money to see how much I have."—*Medical and Surgical Reporter.*

---

The Provincial Board of Health, owing to the unsatisfactory character of the imported vaccine points, has decided to request the Government to establish a vaccine farm in the Province of Ontario.

